# Relationship between Fear-Avoidance Beliefs and Reaction Time Changes Prior to and following Exercise-Induced Muscle Fatigue in Chronic Low Back Pain

**DOI:** 10.1155/2024/9982411

**Published:** 2024-01-27

**Authors:** Wenwu Xiao, Huaichun Yang, Zengming Hao, Menglin Li, Mengchu Zhao, Siyun Zhang, Guifang Zhang, Haian Mao, Chuhuai Wang

**Affiliations:** ^1^Department of Rehabilitation Medicine, The First Affiliated Hospital of Sun Yat-Sen University, Guangzhou 510080, China; ^2^Department of Rehabilitation, Guangzhou First People's Hospital, School of Medicine, South China University of Technology, Guangzhou, Guangdong 510180, China; ^3^Department of Rehabilitation Sciences, The Hong Kong Polytechnic University, Hong Kong, SAR 999077, China

## Abstract

**Background:**

Reaction time is a reliable indicator of the velocity and efficiency of neuromuscular control and may be associated with fear-avoidance beliefs. However, the effect of exercise-induced muscle fatigue on reaction time in chronic low back pain (cLBP) and its relationship with fear-avoidance beliefs remains poorly understood.

**Objectives:**

This study aimed to reveal the relationship between fear-avoidance beliefs and reaction time changes before and after exercise-induced muscle fatigue in cLBP.

**Methods:**

Twenty-five patients with cLBP were tested by the Biering–Sorensen test (BST) to induce exhaustive muscle fatigue. Total reaction time (TRT), premotor time (PMT), and electromechanical delay (EMD) of dominated deltoid muscle were recorded by surface electromyography during the arm-raising task with visual cues before and after muscle fatigue. The mean difference (MD) of TRT (MD_TRT_), PMT (MD_PMT_), and EMD (MD_EMD_) was calculated from the changes before and after muscle fatigue. Fear-avoidance beliefs questionnaire (FABQ) was applied to evaluate fear-avoidance beliefs before muscle fatigue. In addition, the duration time of BST was recorded for each subject.

**Results:**

TRT and PMT of dominated deltoid muscle were prolonged after exercise-induced muscle fatigue (*Z* = 3.511, *p* < 0.001; *t* = 3.431, *p* = 0.001), while there was no statistical difference in EMD (*Z* = 1.029, *p* = 0.304). Correlation analysis showed that both the MD_TRT_ and MD_PMT_ were positively correlated with FABQ (*r* = 0.418, *p* = 0.042; *r* = 0.422, *p* = 0.040).

**Conclusions:**

These findings suggested that we should pay attention to both muscle fatigue-induced reaction time delay in cLBP management and the possible psychological mechanism involved in it. Furthermore, this study implied that FABQ-based psychotherapy might serve as a potential approach for cLBP treatment by improving reaction time delay. This trial is registered with ChiCTR2300074348.

## 1. Introduction

Chronic low back pain (cLBP) is a major global challenge to the world and it contributes to people's years lived with disability (YLDs) [1], yet up to 85% of patients with cLBP are nonspecific [2, 3]. Since the pathogenesis of cLBP is still unclear and there is a lack of targeted treatment, the therapeutical benefit is often temporary and the long-term efficacy is unsatisfactory. Therefore, it is imperative to pay more attention to cLBP and delve deeper into its potential risk factors and abnormal changes.

Reaction time refers to the time window that the body responds to postural perturbation [4, 5], which is consisted of premotor time (PMT) and electromechanical delay (EMD) [6], and can be affected by physical function [7], aging [8], motor and cognitive [9], gender [10], metabolic [11], psychology [12], and fatigue [13]. PMT represents the efficiency of neuromuscular response to disturbance, reflecting cognitive and psychological function. PMT also shows a positive correlation with the fear-avoidance beliefs questionnaire (FABQ) [14]. EMD represents the time interval from muscle electrical signal activation to muscle mechanical contraction [15]. In terms of the whole neuromuscular response process, the total reaction time (TRT) consists of PMT and EMD. In general, PMT depends on the function of the central nervous system, while EMD depends on the morphological structure and functional status of the peripheral tissues. Investigating these phases separately might further elucidate the onset changes in neuromuscular response processes under various internal and external environmental alteration conditions, which could be applied to research areas such as physical exercise [16], sports injuries [17, 18], chronic pain [18, 19], and fatigue [20–22].

Exercise-induced muscle fatigue refers to the reduction in expected muscle strength and force output following repetitive muscle contractions caused by physical activity [20, 23], which has been found to be closely associated with cLBP. Studies have reported that cLBP patients often experience exercise-induced muscle fatigue, and long-term sedentary posture or heavy work loading can contribute to the occurrence or recurrence of cLBP [24, 25]. Recent studies suggest that postural control alteration may be a common characteristic of individuals with cLBP when faced with internal or external perturbation [26, 27]. Exercise-induced muscle fatigue can affect sensory input, motor output, and neuromuscular control, thereby influencing postural control [28, 29]. Previous studies have clarified that cLBP patients exhibit a retarded reaction time compared to asymptomatic individuals [30, 31]. For instance, Sipko et al. [32] found that cLBP patients tend to adopt compensation strategies through the sit-to-stand (STS) test, i.e., shorter preparation time and longer postural stabilization time, compared to healthy individuals. Additionally, high levels of pain were associated with avoidance behaviors during STS execution, leading to decreased ground reaction peak force and increased time to peak. Furthermore, the severity of pain was found to be correlated with delayed muscle reaction time [19].

CLBP is often associated with psychosocial disorders, such as anxiety, depression, and fear of painful activities [33, 34]. The fear-avoidance beliefs questionnaire (FABQ) is widely recognized as a common psychological assessment tool for evaluating fear-avoidance beliefs related to pain [35]. Notably, research has demonstrated a potential correlation between fear avoidance and the efficacy of neuromuscular response [36]. Individuals with cLBP often exhibit fear-avoidance attitudes towards daily activities as a result of their persistent pain symptoms [35], which may impact the speed and efficiency of neuromuscular responses, as well as postural control. Therefore, investigating the potential link between FABQ and altered reaction time could contribute to a deeper understanding of the postural control changes in cLBP. However, evidence on the effect of exercise-induced muscle fatigue on the TRT and its components (PMT and EMD) under the internal perturbation in cLBP patients is lacking. It is also unclear whether changes in reaction time of neuromuscular control processes caused by exercise-induced muscle fatigue are related to fear-avoidance beliefs in individuals with cLBP.

Based on the abovementioned studies, we propose the following hypothesis: exercise-induced muscle fatigue may cause changes in reaction time of neuromuscular control in cLBP patients, and these changes may be correlated with fear-avoidance beliefs. In the present study, surface electromyography (s-EMG) was deployed to detect the TRT, PMT, and EMD during the arm-raising task (ART) prior to and following exercise-induced muscle fatigue, and the FABQ was evaluated to assess fear-avoidance beliefs. Through these endeavors, this study would help to understand the possible mechanism of reaction time change in postural control of cLBP induced by muscle fatigue and its relationship with fear-avoidance beliefs. Besides, it also provides potential insights into the psychological interventions for cLBP.

## 2. Material and Methods

### 2.1. Population and Study Design

This was a cross-sectional study, which was approved by the Ethics Committee of the First Affiliated Hospital of Sun Yat-sen University (No. [2023]386-1) and registered in the China Clinical Trial Registry Center (No. ChiCTR2300074348). Twenty-five cLBP patients were recruited in this study, and the Helsinki Declaration was considered. All subjects were fully informed of the experimental process and potential risks such as electrode pad allergy and falls and signed informed consent. The s-EMG was applied to measure the reaction time of all subjects during ART before and after exercise-induced muscle fatigue. Basic information about the subjects such as name, age, gender, weight, height, body mass index (BMI), visual analogue scale (VAS) [37], and FABQ [35] was collected before the test.

### 2.2. Sample Size Calculation

The sample size in this study was computed by G^*∗*^Power software (version 3.1.9.4, Kiel, Germany). We applied the TRT parameter changes (before vs after muscle fatigue, 0.472 ± 0.186 vs 0.539 ± 0.152) of eight subjects in the preliminary study to conduct the sample calculation by G-power, setting *α* = 0.05, power (1-*β*) = 0.8, and effect size = 0.67. Meanwhile, the effect size of 0.67 was determined by the mean difference changes of TRT before and after muscle fatigue. The total sample size was 20 for this research. Considering the dropout rate of approximately 20%, we planned to recruit 25 participants.

### 2.3. Enrollment Procedure

The diagnosis of cLBP was made by a professional physician according to the guidelines and management of cLBP [38, 39]. The inclusion and exclusion criteria were modified slightly according to the previous studies [40, 41]. The inclusion criteria for cLBP were as follows: (1) aged between 18∼59 years; (2) persistent or recurrent low back pain symptoms for more than 3 months, with at least one recurrent low back pain in the past year; (3) the right dominant hand [42] with normal upper extremity function; (4) pain location concentrated between the 12th rib and the gluteus sulcus and VAS score greater than 3 points according to the VAS; (5) no clear cause of low back pain. The exclusion criteria referred to the following tips: (1) participants who had a history of pelvic or spinal surgery; (2) any specific lumbar pathological changes, such as lumbar sprain, scoliosis, spinal tumors, vertebral fractures, lumbar spinal stenosis or disc herniation, osteoporosis, obligatory spondylitis, osteoarthritis, and lumbar tuberculosis.; (3) patients with the symptoms of extremity nerve root or nerve radiation; (4) participants with BMI over 31 kg/m^2^ or pregnant; (5) subjects who received systematic physical rehabilitation therapy in the past 3 months; (6) subjects with severe audiovisual dysfunction; (7) participants with severe cognitive impairment, depression, or anxiety who are unable to cooperate with the test.

### 2.4. Internal Perturbation Task

The ART, a reliable method inducing internal postural perturbation, was adopted in our study according to a previous study [43]. The task procedure was as follows: the subjects stood on a force platform. A visual arm-raising signal (depicted as a solid black circle) was presented on the monitor placed 2 meters in front of subjects at the eye level. The signal was presented three times in 30 s randomly. Once it arose, the subject was instructed to raise their right arm to the shoulder plane as quickly and explosively as possible and return to the neutral position when the signal disappeared after approximately 5 s. In addition, subjects were allowed to practice three times to ensure they were familiar with the test procedure.

### 2.5. Muscle Fatigue Test

The Biering–Sorensen test (BST) is a valuable protocol to induce muscle fatigue for low back pain patients [44, 45]. The workflow was as follows: subjects were positioned in a prone lying position on a therapy bed with the upper edge of the iliac crest aligned with the boundary of the bed, and their arms were crossed over their chest. Belts were used to secure the lower part of the body at the hip, popliteal, and ankle levels. Once the test commenced, subjects were instructed to maintain their body's longitudinal axis on a horizontal line as long as possible to induce exhaustion. The test would be terminated if the subjects were unable to overcome the force of gravity, maintain a horizontal position after several reminders, or experience pain [46, 47]. We measured the subjects' endurance by recording the duration time of the Biering–Sorensen test (BST-DT). Following the test, we promptly conducted the ART within a 20-second time frame [29] and obtained s-EMG signals.

### 2.6. Reaction Time Detection

The s-EMG is a common method for measuring muscle reaction time [48], which can effectively detect PMT and EMD, respectively [6, 49]. In this study, we used wireless s-EMG equipment (Trigno™, Delsys Incorporated, USA) for reaction time detection during an internal postural perturbation task. The wireless s-EMG electrode was placed on the dominant anterior deltoid muscle of the subject. The time point when the screen displays black dots (visual cue for arm raising) was marked as *T*_0_. The subject responded to the visual cue by raising the dominant arm as fast and explosively as possible. The moment when the initial mechanical contraction occurred was detected by the displacement sensor built in the electrode and was denoted as *T*_torque_. The start time of the myoelectrical signal activation was recorded as *T*_electrical_. TRT = *T*_torque_ − *T*_0_, which is the time interval from the time point of the visual cue signal to the start time of mechanical movement of the dominant arm. PMT = *T*_electrical_ − *T*_0_, which is the time interval from the time point of the visual cue signal to the activation time of the deltoid muscle electromyographic signal; EMD = TRT-PMT, which is the time interval between the myoelectrical signal activation to the start of mechanical contraction. In addition, the mean differences of TRT, PMT, and EMD were calculated from the changes before and after fatigue and counted as MD_TRT_, MD_PMT_, and MD_EMD_, respectively. In order to reduce the error, we calculated the average value as the final statistical indicator during the 3 trials of the ART task.

### 2.7. FABQ Scale Assessment

The FABQ is a widely used tool for evaluating the influence of fear-avoidance beliefs on activity and work in patients with low back pain. It has been revealed a good reliability and validity when applied to cLBP [35]. The questionnaire comprises 16 items, with 5 items specifically focusing on the impact of fear-avoidance beliefs on low back pain during physical activity and 11 items assessing the impact on work-related activities. Each item is rated on a 7-point scale ranging from 0 to 6. The total score possible on the questionnaire is 96, with higher scores indicating higher levels of fear-avoidance beliefs. In this trial, we assessed FABQ by electronic questionnaire for all subjects.

### 2.8. Statistical Analysis

MATLAB R2022a software (MathWorks, Natick, MA, USA) was conducted to s-EMG signals analysis. SPSS 20.0 (IBM, Chicago, USA) was used for data entry and statistical analysis. A normality test is performed for all measurement data (age, height, weight, BMI, BST-DT, VAS, FABQ, TRT, PMT, and EMD). The data were expressed as mean ± standard deviation and compared by paired sample *t*-test when the data distributions fit the normal curve. Otherwise, the data were expressed as the median (25%∼75% interquartile range) and compared with nonparametric tests. Pearson's or Spearman's correlation analysis was used to analyze the correlation between the indexes of MD_TRT_, MD_PMT_, MD_EMD_, and BST-DT with FABQ according to the results of the normality test. *p* < 0.05 indicates a statistical difference.

## 3. Results

### 3.1. Test Flowchart and Basic Demographic Data

**Figure 1:**
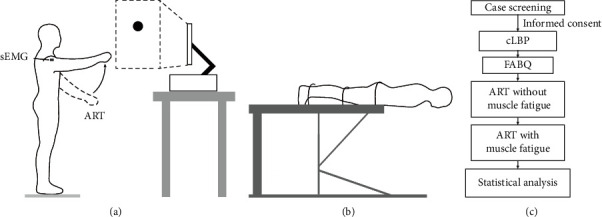
Experimental paradigm and the test flowchart. (a) Schematic diagram of the arm-raising task (ART) with visual cues was illustrated. (b) Schematic diagram of the Biering–Sorenson test (BST). (c) The flowchart of the study.

A total of 25 cLBP patients met the criteria and were finally recruited for this study. The FABQ scores, BST-DT, and the basic demographic characteristics of the enrolled subjects are shown in Table 1. Additionally, Figure 1 shows the relevant experimental paradigm and process of this study.

### 3.2. Change of Reaction Time before and after Muscle Fatigue

As shown in Table 2, by performing internal postural perturbations of the ART with visual cues, the TRT and PMT of cLBP patients after muscle fatigue were prolonged, and the difference was statistically significant (*Z* = 3.511, Cohen's *d* = 1.973, *η*^2^ = 0.493, *p* < 0.001; *Z* = 3.431, Cohen's *d* = 1.887, *η*^2^ = 0.471, *p*=0.001, respectively) compared to the condition before muscle fatigue, but no statistical difference was found in EMD (*Z* = 1.029, Cohen's *d* = 0.421, *η*^2^ = 0.042, *p*=0.304).

### 3.3. Correlation Analysis between Reaction Time Change Degree, BST-DT with FABQ

By calculating the change degree of reaction time before and after muscle fatigue, the mean difference (MD) of TRT, PMT, and EMD could be obtained, respectively, and was noted as MD_TRT_, MD_PMT_, and MD_EMD_. Correlation analysis showed that the MD_TRT_ and MD_PMT_ were positively correlated with FABQ (*r* = 0.418, *p*=0.042; *r* = 0.422, *p*=0.040, respectively), but there was no significant correlation between MD_EMD_ and FABQ (*r* = −0.152, *p*=0.478). In addition, the correlation analysis also showed no correlation between BST-DT and FABQ (*r* = −0.019, *p*=0.930). The details are presented in Table 3.

## 4. Discussion

In this study, we used the BST to induce muscle fatigue in patients with cLBP, performed ART with visual stimulation before and after muscle fatigue, and used s-EMG to analyze the neuromuscular reaction time during this process. We found that (1) the TRT and PMT were prolonged after muscle fatigue in cLBP, compared with before muscle fatigue; (2) correlation analysis revealed that the change degree of MD_TRT_ and MD_PMT_ was positively correlated with fear-avoidance beliefs. This study suggested that the reaction time of neuromuscular control processes would be altered by muscle fatigue in patients with cLBP and that these changes were associated with fear-avoidance beliefs.

### 4.1. Muscle Fatigue and Reaction Time

Previous studies have shown that cLBP patients are more prone to muscle fatigue than the asymptomatic population [50–52]. Behaviors such as longtime sitting or standing are important factors for the occurrence or recurrence of cLBP [53, 54]. Studies have found that muscle fatigue can induce changes in postural control in patients with cLBP [46, 55]. For example, cLBP patients had significantly decreased sagittal spine stability compared with healthy subjects when performing repeated uplifting as a muscle fatigue-inducing protocol [56]. Besides, cLBP exhibited relatively more unpredictable lumbar spine motion after muscle fatigue compared to the matched healthy subjects [57]. The TRT, an important indicator of neuromuscular control ability [58, 59], is fractioned into two components, i.e., PMT and EMD. The PMT contains cognitive components, while EMD indicates pure electromechanical delay [60, 61]. However, the effect of muscle fatigue on the reaction time of limb movements in cLBP was reported seldomly [18]. To address this issue, this study explored the potential relationship between muscle fatigue and reaction time through the arm-raising task with visual cues prior to and following exercise-induced muscle fatigue in patients with cLBP and quantified the weight of PMT and EMD in TRT. Notably, the reaction time in this study could be accurately measured by a technical modification that allows the visual stimulus presentation to be fully synchronized with the s-EMG acquisition. To the best of our knowledge, this elimination of absolute errors in reaction time acquisition has not been explicitly reported in previous studies.

A study conducted by Abdollahi et al. demonstrated that basketball players with cLBP have prolonged EMD in gastrocnemius and tibialis anterior muscles and shortened EMD of semitendinosus, vastus lateralis, and vastus medialis oblique muscles after lower limb muscle fatigue than healthy basketball players [18]. Another study conducted by Le Mansec et al. found that EMD of the biceps brachii in healthy individuals would be increased after muscle fatigue induced by intermittent contractions of biceps brachii [20]. Echoing previous studies, our study found that trunk muscle fatigue induced TRT and PMT delay in deltoid muscle, but not EMD. Regarding the muscle fatigue methods and muscle observations in our study, the deltoid muscles of the subjects were undisturbed by fatigue load. EMD is defined as the time interval from the activation time of electromyographic signals to the contraction time of the target muscle, which is more closely related to local tissue metabolism [62]. Thus, these might help explaining why EMD in deltoid muscle was not affected in our results, which was the reason for these discrepant results between our study and previous studies. However, although the deltoid muscle has not suffered from fatigue, we still observed the delay of TRT and PMT. This suggested that the delay of TRT and PMT of the deltoid muscle might be due to the changes in cognitive and psychological activities. The delay of TRT is mainly dependent on the delay of PMT, which indicates that PMT weighs more than EMD in the whole reaction time. The result is consistent with the observation reported by Le Mansec et al. [20].

### 4.2. Fear-Avoidance Beliefs and Reaction Time

Reaction time and its main component, PMT, are reported to be strongly correlated with cognitive-mental activity [63, 64]. Patients with cLBP tend to have a certain degree of fear-avoidance beliefs due to long-term pain [65]. A previous study by performing auditory reaction time tasks while administering electrical stimulation on the arm or back demonstrated that cLBP patients with higher pain fear would be accompanied by a more obvious reaction time delay [66]. However, to our knowledge, whether the TRT and/or PMT delay induced by muscle fatigue is related to fear-avoidance beliefs in cLBP patients has not been clearly reported.

In this study, we performed a correlation analysis by assessing FABQ scores and the reaction time changes before and after exercise-induced muscle fatigue in cLBP patients, the results showed that TRT and PMT delays were positively correlated with FABQ scores. Additionally, although the deltoid muscle was not directly affected by fatigue load in the present study, a delay in its reaction time was still observed. Therefore, it is reasonable to assume that this reaction time delay is related to psychological alterations induced by fear-avoidance beliefs. Furthermore, we explored the potential association between fear-avoidance beliefs and task duration time. A previous study by Vincent et al. [67] deemed that fear-avoidance beliefs did not impact walking endurance time in individuals with cLBP. Consistent with these findings, our study also revealed no correlation between FABQ and BST-DT. This might be attributed to the fact that the participants included in this study were relatively young and usually exhibited better self-efficacy. They tended to perceive difficult tasks as a challenge rather than as a danger to avoid [68].

### 4.3. Study Limitations

This study indicated that exercise-induced muscle fatigue contributes to delays of TRT and PMT in individuals with cLBP. These reaction time delays were closely associated with FABQ scores. However, this study did not include healthy subjects and was not able to compare the effect of exercise-induced muscle fatigue on reaction time in healthy populations. Future studies should include healthy individuals for comparison to better elucidate the potential relationship among exercise-induced muscle fatigue, reaction time, and low back pain. Furthermore, considering the close relationship between pain levels and fear-avoidance beliefs, it is necessary to point out that a subset of the subjects in this study with low VAS scores also exhibited relatively weak fear-avoidance beliefs. This could potentially introduce bias into the results. Future research specifically focused on grading pain levels would further enhance the credibility of the findings. Additionally, the study did not investigate the effect of exercise-induced muscle fatigue on other types of reaction time, such as choice reaction time and discrimination reaction time. Future research should aim to explore and compare the effects of exercise-induced muscle fatigue on the aforementioned reaction time, which could shed light on the underlying mechanisms of reaction time alteration in individuals with cLBP.

## 5. Conclusions

The present study demonstrated that exercise-induced muscle fatigue could evoke reaction time alteration in the neuromuscular control processes of cLBP. It shed light on the potential relationship between muscle fatigue and low back pain. Additionally, we found a correlation between this reaction time delay and the FABQ scores, indicating psychological function may be involved in posture control of cLBP, and FABQ-based psychotherapy might be a promising therapeutical approach to cLBP management.

## Figures and Tables

**Table 1:** Demographic variables of the subject's data.

	cLBP (*n* = 25)
Gender (male/female)^a^	10/15
Age (years)^c^	31.56 (25.00, 36.00) [23.00, 56.00]^d^
Weight (kg)^b^	58.72 ± 10.29 [36.00, 76.00]^d^
Height (m)^b^	1.66 ± 0.08 [1.53, 1.80]^d^
BMI (kg/m^2^)^b^	21.17 ± 2.55 [14.79, 26.93]^d^
VAS (score)^b^	5.28 ± 1.62 [3.00, 9.00]^d^
FABQ (score)^b^	47.33 ± 18.43 [6.00, 79.00]^d^
BST-DT (s)^b^	105.08 ± 36.83 [48.00, 220.00]^d^

*Notes*. ^a^Value indicated sex composition ratio. ^b^Value indicated mean ± standard deviation. ^c^Value indicated median (25%∼75% interquartile range). ^d^Value indicated [min, max]. FABQ, fear-avoidance beliefs questionnaire; BST-DT, duration time of the Biering–Sorensen test.

**Table 2:** Changes of TRT, PMT, and EMD before and after exercise-induced muscle fatigue (*n* = 25, s).

	Before MF	After MF	95% CI	Cohen's *d*	*η * ^2^	*Z*	*p* value
TRT	0.358 (0.326, 0.415)	0.411 (0.353, 0.469)	0.026∼0.097	1.973	0.493	3.511	<0.001^*∗*^
PMT	0.333 (0.301, 0.390)	0.386 (0.328, 0.444)	0.024∼0.096	1.887	0.471	3.431	0.001^*∗*^
EMD	0.025 (0.025, 0.026)	0.025 (0.025, 0.027)	0.000∼0.003	0.421	0.042	1.029	0.304

*Notes*. ^*∗*^*p* < 0.05. TRT: total reaction time; PRT: premotor time; EMD: electromechanical delay; *η*^2^: eta squared; MF: muscle fatigue.

**Table 3:** Correlation analysis of MD_TRT_, MD_PMT_, MD_EMD_, and BST-DT with FABQ.

	MD_TRT_		MD_PMT_		MD_EMD_		BST-DT	
	*r* value	*p* value	*r* value	*p* value	*r* value	*p* value	*r* value	*p* value
FABQ	0.418	0.042^*∗*^	0.422	0.040^*∗*^	−0.152	0.478	−0.019	0.930

*Notes*. ^*∗*^*p* < 0.05. MD_TRT_, MD_PMT_, and MD_EMD_ were analyzed by Spearman's correlation analysis with FABQ, respectively. BST-DT with FABQ was analyzed by Pearson's correlation analysis. MD_TRT_: the mean difference of total reaction time; MD_PMT_: the mean difference of premotor time; MD_EMD_: the mean difference of electromechanical delay; FABQ: fear-avoidance beliefs questionnaire; BST-DT: duration time of the Biering–Sorensen test.

## Data Availability

The data are available from the corresponding author upon reasonable request.

## Additional Points

*Clinical Trial Registration Number* registered in the China Clinical Trial Registry Center (No. ChiCTR2300074348).

## References

[1] Clark S., Horton R. (2018). Low back pain: a major global challenge. *The Lancet*.

[2] Sanzarello I., Merlini L., Rosa M. A. (2016). Central sensitization in chronic low back pain: a narrative review. *Journal of Back and Musculoskeletal Rehabilitation*.

[3] Hooten W. M., Cohen S. P. (2015). Evaluation and treatment of low back pain: a clinically focused review for primary care specialists. *Mayo Clinic Proceedings*.

[4] Peters M. L., Vlaeyen J. W. S., van Drunen C. (2000). Do fibromyalgia patients display hypervigilance for innocuous somatosensory stimuli? Application of a body scanning reaction time paradigm. *Pain*.

[5] Aziz-Zadeh L., Iacoboni M., Zaidel E. (2006). Hemispheric sensitivity to body stimuli in simple reaction time. *Experimental Brain Research*.

[6] Szpala A., Rutkowska-Kucharska A. (2017). Electromechanical response times in the knee muscles in young and old women. *Muscle & Nerve*.

[7] Jimenez-Garcia J. D., Martinez-Amat A., Hita-Contreras F., Fabrega-Cuadros R., Alvarez-Salvago F., Aibar-Almazan A. (2021). Muscle strength and physical performance are associated with reaction time performance in older people. *International Journal of Environmental Research and Public Health*.

[8] Johari K., den Ouden D. B., Behroozmand R. (2018). Effects of aging on temporal predictive mechanisms of speech and hand motor reaction time. *Aging Clinical and Experimental Research*.

[9] Villa-Sanchez B., Emadi Andani M., Cesari P., Fiorio M. (2021). The effect of motor and cognitive placebos on the serial reaction time task. *European Journal of Neuroscience*.

[10] Agustiningsih D., Sofyana M., Budiharjo S. (2021). Reaction times among batik workers: the influence of gender and occupational lead exposure. *International Journal of Environmental Research and Public Health*.

[11] Parker K. L., Alberico S. L., Miller A. D., Narayanan N. S. (2013). Prefrontal D1 dopamine signaling is necessary for temporal expectation during reaction time performance. *Neuroscience*.

[12] Kang I., Molenaar D., Ratcliff R. (2023). A modeling framework to examine psychological processes underlying ordinal responses and response times of psychometric data. *Psychometrika*.

[13] Sant’Ana J., Franchini E., da Silva V., Diefenthaeler F. (2017). Effect of fatigue on reaction time, response time, performance time, and kick impact in taekwondo roundhouse kick. *Sports Biomechanics*.

[14] Genoese F., Baez S. E., Heebner N., Hoch M. C., Hoch J. M. (2021). The relationship between injury-related fear and visuomotor reaction time in individuals with a history of anterior cruciate ligament reconstruction. *Journal of Sport Rehabilitation*.

[15] Lacourpaille L., Hug F., Nordez A. (2013). Influence of passive muscle tension on electromechanical delay in humans. *PLoS One*.

[16] Mero A., Komi P. V. (1990). Reaction time and electromyographic activity during a sprint start. *European Journal of Applied Physiology and Occupational Physiology*.

[17] Besier T. F., Lloyd D. G., Ackland T. R. (2003). Muscle activation strategies at the knee during running and cutting maneuvers. *Medicine & Science in Sports & Exercise*.

[18] Abdollahi S., Sheikhhoseini R., Roostayi M. M., Huddleston W. E. (2022). The effect of fatigue on electromechanical response times in basketball players with and without persistent low back pain. *Scientific Reports*.

[19] Luoto S., Alaranta H., Taimela S., Hurri H. (1998). Psychomotor speed in chronic low-back pain patients and healthy controls: construct validity and clinical significance of the measure. *Perceptual and Motor Skills*.

[20] Le Mansec Y., Dorel S., Nordez A., Jubeau M. (2019). Is reaction time altered by mental or physical exertion?. *European Journal of Applied Physiology*.

[21] Soto-Leon V., Alonso-Bonilla C., Peinado-Palomino D. (2020). Effects of fatigue induced by repetitive movements and isometric tasks on reaction time. *Human Movement Science*.

[22] Wilder D. G., Aleksiev A. R., Magnusson M. L., Pope M. H., Spratt K. F., Goel V. K. (1996). Muscular response to sudden load. A tool to evaluate fatigue and rehabilitation. *Spine*.

[23] Knicker A. J., Renshaw I., Oldham A. R., Cairns S. P. (2011). Interactive processes link the multiple symptoms of fatigue in sport competition. *Sports Medicine*.

[24] Kumamoto T., Seko T., Matsuda R., Miura S. (2021). Repeated standing back extension exercise: influence on muscle shear modulus change after lumbodorsal muscle fatigue. *Work*.

[25] Jubany J., Danneels L., Angulo-Barroso R. (2017). The influence of fatigue and chronic low back pain on muscle recruitment patterns following an unexpected external perturbation. *BMC Musculoskeletal Disorders*.

[26] Knox M. F., Chipchase L. S., Schabrun S. M., Romero R. J., Marshall P. W. M. (2018). Anticipatory and compensatory postural adjustments in people with low back pain: a systematic review and meta-analysis. *The Spine Journal*.

[27] Yu Q., Huo Y., Chen M. (2021). A study on the relationship between postural control and pain-related clinical outcomes in patients with chronic nonspecific low back pain. *Pain Research and Management*.

[28] Harkins K. M., Mattacola C. G., Uhl T. L., Malone T. R., McCrory J. L. (2005). Effects of 2 ankle fatigue models on the duration of postural stability dysfunction. *Journal of Athletic Training*.

[29] Hsu W. L., Chen C. P., Nikkhoo M. (2020). Fatigue changes neck muscle control and deteriorates postural stability during arm movement perturbations in patients with chronic neck pain. *The Spine Journal*.

[30] Taimela S., Osterman K., Alaranta H., Soukka A., Kujala U. M. (1993). Long psychomotor reaction time in patients with chronic low-back pain: preliminary report. *Archives of Physical Medicine and Rehabilitation*.

[31] Kusters D., Vollenbroek-Hutten M. M., Hermens H. J. (2011). Motor performance in chronic low back pain: is there an influence of pain-related cognitions? A pilot study. *BMC Musculoskeletal Disorders*.

[32] Sipko T., Glibowski E., Barczyk-Pawelec K., Kuczynski M. (2016). The effect of chronic pain intensity on sit-to-stand strategy in patients with herniated lumbar disks. *Journal of Manipulative and Physiological Therapeutics*.

[33] Bletzer J., Gantz S., Voigt T., Neubauer E., Schiltenwolf M. (2017). Chronic low back pain and psychological comorbidity. *Schmerz, Der*.

[34] Moore J. E. (2010). Chronic low back pain and psychosocial issues. *Physical Medicine and Rehabilitation Clinics of North America*.

[35] Waddell G., Newton M., Henderson I., Somerville D., Main C. J. (1993). A Fear-Avoidance Beliefs Questionnaire (FABQ) and the role of fear-avoidance beliefs in chronic low back pain and disability. *Pain*.

[36] Dubois J. D., Abboud J., St-Pierre C., Piche M., Descarreaux M. (2014). Neuromuscular adaptations predict functional disability independently of clinical pain and psychological factors in patients with chronic non-specific low back pain. *Journal of Electromyography and Kinesiology*.

[37] Boonstra A. M., Schiphorst Preuper H. R., Reneman M. F., Posthumus J. B., Stewart R. E. (2008). Reliability and validity of the visual analogue scale for disability in patients with chronic musculoskeletal pain. *International Journal of Rehabilitation Research*.

[38] Koes B. W., van Tulder M. W., Thomas S. (2006). Diagnosis and treatment of low back pain. *BMJ*.

[39] Chou R., Qaseem A., Snow V. (2007). Diagnosis and treatment of low back pain: a joint clinical practice guideline from the American college of physicians and the American pain society. *Annals of Internal Medicine*.

[40] Bardin L. D., King P., Maher C. G. (2017). Diagnostic triage for low back pain: a practical approach for primary care. *Medical Journal of Australia*.

[41] Xiao W., Zheng F., Dong K., Wang Z., Zu Y., Wang C. (2022). Ultrasonography comparison of diaphragm morphological structure and function in young and middle-aged subjects with and without non-specific chronic low back pain: a case-control study. *Pain Research and Management*.

[42] Paracchini S., Scerri T., Rogers L. J., Vallortigara G. (2017). Genetics of human handedness and laterality. *Lateralized Brain Functions: Methods in Human and Non-human Species*.

[43] Ledebt A., Savelsbergh G. J. (2014). Postural adaptation during arm raising in children with and without unilateral cerebral palsy. *Research in Developmental Disabilities*.

[44] Villafane J. H., Gobbo M., Peranzoni M. (2016). Validity and everyday clinical applicability of lumbar muscle fatigue assessment methods in patients with chronic non-specific low back pain: a systematic review. *Disability & Rehabilitation*.

[45] Coorevits P., Danneels L., Cambier D., Ramon H., Vanderstraeten G. (2008). Assessment of the validity of the Biering-Sorensen test for measuring back muscle fatigue based on EMG median frequency characteristics of back and hip muscles. *Journal of Electromyography and Kinesiology*.

[46] Johanson E., Brumagne S., Janssens L., Pijnenburg M., Claeys K., Paasuke M. (2011). The effect of acute back muscle fatigue on postural control strategy in people with and without recurrent low back pain. *European Spine Journal*.

[47] Latimer J., Maher C. G., Refshauge K., Colaco I. (1999). The reliability and validity of the Biering-Sorensen test in asymptomatic subjects and subjects reporting current or previous nonspecific low back pain. *Spine*.

[48] Arnold P., Vantieghem S., Gorus E. (2015). Age-related differences in muscle recruitment and reaction-time performance. *Experimental Gerontology*.

[49] Ayala F., De Ste Croix M., Sainz de Baranda P., Santonja F. (2014). Inter-session reliability and sex-related differences in hamstrings total reaction time, pre-motor time and motor time during eccentric isokinetic contractions in recreational athlete. *Journal of Electromyography and Kinesiology*.

[50] Correia J. P., Oliveira R., Vaz J. R., Silva L., Pezarat-Correia P. (2016). Trunk muscle activation, fatigue and low back pain in tennis players. *Journal of Science and Medicine in Sport*.

[51] Janssens L., Brumagne S., McConnell A. K., Hermans G., Troosters T., Gayan-Ramirez G. (2013). Greater diaphragm fatigability in individuals with recurrent low back pain. *Respiratory Physiology & Neurobiology*.

[52] da Silva R. A., Vieira E. R., Cabrera M. (2015). Back muscle fatigue of younger and older adults with and without chronic low back pain using two protocols: a case-control study. *Journal of Electromyography and Kinesiology*.

[53] Baradaran Mahdavi S., Riahi R., Vahdatpour B., Kelishadi R. (2021). Association between sedentary behavior and low back pain; A systematic review and meta-analysis. *Health Promotion Perspectives*.

[54] Gallagher K. M., Callaghan J. P. (2015). Early static standing is associated with prolonged standing induced low back pain. *Human Movement Science*.

[55] Tajali S., Roozbehfar N., Mehravar M., Goharpey S., Gayem K. (2022). Effects of back extensor and hip abductor fatigue on dynamic postural stability in patients with nonspecific chronic low back pain: a case-control study. *Physiotherapy Theory and Practice*.

[56] Asgari N., Sanjari M. A., Esteki A. (2017). Local dynamic stability of the spine and its coordinated lower joints during repetitive Lifting: effects of fatigue and chronic low back pain. *Human Movement Science*.

[57] Bauer C. M., Rast F. M., Ernst M. J. (2017). The effect of muscle fatigue and low back pain on lumbar movement variability and complexity. *Journal of Electromyography and Kinesiology*.

[58] Richie D. H. (2001). Functional instability of the ankle and the role of neuromuscular control: a comprehensive review. *The Journal of Foot & Ankle Surgery*.

[59] Morcelli M. H., LaRoche D. P., Crozara L. F. (2016). Neuromuscular performance in the hip joint of elderly fallers and non-fallers. *Aging Clinical and Experimental Research*.

[60] Holl N., Wuebbenhorst K., Behrens M., Zschorlich V. (2015). The effect of age on coordination of stabilization during changing environmental dynamics. *Brain Research*.

[61] Le Mansec Y., Nordez A., Dorel S., Jubeau M. (2018). Reaction time can be measured during voluntary contractions with electrode array. *Clinical Physiology and Functional Imaging*.

[62] Constantino J., Hu Y., Lardo A. C., Trayanova N. A. (2013). Mechanistic insight into prolonged electromechanical delay in dyssynchronous heart failure: a computational study. *American Journal of Physiology-Heart and Circulatory Physiology*.

[63] Jakobsen L. H., Sorensen J. M., Rask I. K., Jensen B. S., Kondrup J. (2011). Validation of reaction time as a measure of cognitive function and quality of life in healthy subjects and patients. *Nutrition*.

[64] J Alibazi R., Kidgell D., Zoghi M., Jaberzadeh S. (2020). What are the acute effects of aerobic exercise on fractionated response time: a systematic review and meta-analysis. *Journal of Science in Sport and Exercise*.

[65] Wertli M. M., Rasmussen-Barr E., Weiser S., Bachmann L. M., Brunner F. (2014). The role of fear avoidance beliefs as a prognostic factor for outcome in patients with nonspecific low back pain: a systematic review. *The Spine Journal*.

[66] Peters M. L., Vlaeyen J. W., Kunnen A. M. (2002). Is pain-related fear a predictor of somatosensory hypervigilance in chronic low back pain patients?. *Behaviour Research and Therapy*.

[67] Vincent H. K., Seay A. N., Montero C., Conrad B. P., Hurley R. W., Vincent K. R. (2013). Kinesiophobia and fear-avoidance beliefs in overweight older adults with chronic low-back pain: relationship to walking endurance--part II. *American Journal of Physical Medicine & Rehabilitation*.

[68] Bandura A. (1995). *Self-efficacy in Changing Societies*.

